# An imbalanced parental genome ratio affects the development of rice zygotes

**DOI:** 10.1093/jxb/ery094

**Published:** 2018-03-10

**Authors:** Erika Toda, Yukinosuke Ohnishi, Takashi Okamoto

**Affiliations:** 1 Department of Biological Sciences, Tokyo Metropolitan University, Minami-osawa, Hachioji, Tokyo, Japan; 2 Plant Breeding Innovation Laboratory, RIKEN Innovation Center, Tsurumi-ku, Yokohama, Japan

**Keywords:** Egg cell, *Oryza sativa*, parental balance, polyploidy, sperm cell, zygote, zygotic development

## Abstract

Upon double fertilization, one sperm cell fuses with the egg cell to form a zygote with a 1:1 maternal-to-paternal genome ratio (1m:1p), and another sperm cell fuses with the central cell to form a triploid primary endosperm cell with a 2m:1p ratio, resulting in formation of the embryo and the endosperm, respectively. The endosperm is known to be considerably sensitive to the ratio of the parental genomes. However, the effect of an imbalance of the parental genomes on zygotic development and embryogenesis has not been well studied, because it is difficult to reproduce the parental genome-imbalanced situation in zygotes and to monitor the developmental profile of zygotes without external effects from the endosperm. In this study, we produced polyploid zygotes with an imbalanced parental genome ratio by electro-fusion of isolated rice gametes and observed their developmental profiles. Polyploid zygotes with an excess maternal gamete/genome developed normally, whereas approximately half to three-quarters of polyploid zygotes with a paternal excess showed developmental arrests. These results indicate that paternal and maternal genomes synergistically serve zygote development with distinct functions, and that genes with monoallelic expression play important roles during zygotic development and embryogenesis.

## Teaser

Zygotes with excess paternal genome show arrest at the developmental step of first division, whereas zygotes with excess maternal genome develop normally.

## Introduction

In angiosperms, the sporophytic generation is initiated by double fertilization to form seeds (Raghavan, 2003). Upon double fertilization, two sperm cells are delivered into an embryo sac via a pollen tube. One sperm cell fuses with the egg cell to form a zygote with a 1:1 maternal-to-paternal genome ratio (1m:1p), and the other sperm cell fuses with the central cell to form a triploid primary endosperm cell with a 2m:1p ratio. These zygote and primary endosperm cells develop, respectively, into the embryo that transmits genetic material from the parents to the next generation and the endosperm that nourishes the developing embryo and seedling (Nawaschin, 1898; Guignard, 1899; Russell, 1992).

A seed consists of three tissues with different parental genomic content: embryo (1m:1p), endosperm (2m:1p), and maternal seed coat (2m:0p). Of the three tissues, the endosperm is known to be considerably sensitive to ploidy differences between parents (Haig and Westoby, 1991). In Arabidopsis seeds prepared by paternal-excess crosses between diploid and tetraploid plants (2*x* female×4*x* male), cellularization of endosperm was delayed, and the size of seeds was enlarged. However, maternal-excess crosses (4*x* female×2*x* male) promoted precocious endosperm cellularization and produced small seeds (Scott *et al.*, 1998; Tiwari *et al.*, 2010; Lu *et al.*, 2012). Furthermore, reciprocal crosses between diploid and hexaploid plants (2*x*×6*x* and 6*x*×2*x*) showed a more extreme phenotype than those between diploid and tetraploid plants, and resulted in seed abortion with only small and shriveled seeds (Scott *et al.*, 1998). The effects of ploidy differences in parents on endosperm development were also investigated in rice, and similar cellularization characteristics of endosperm was observed (Sekine *et al.*, 2013). The effects of parental imbalance on endosperm development have been partly explained by epigenetic regulation of the parental genome (Feil and Berger, 2007).

As for embryo development, Arabidopsis embryos derived from reciprocal crosses between diploid and hexaploid plants aborted at the globular to heart stage (Scott *et al.*, 1998). In rice, swollen embryos were observed in seeds from the 4*x*×2*x* maternal-excess cross, and no embryo-like structures were detected in seeds from the 2*x*×4*x* paternal-excess cross (Sekine *et al.*, 2013). Although development of a zygote also appeared to be affected by interploidy crosses both in Arabidopsis and in rice, it is thought that abnormality in zygotic embryogenesis by an interploid cross is not due to a parental imbalance in zygotes or embryos but to a side effect of the developmental defects of endosperm induced by a parental imbalance, because endosperm functions as a possible growth regulator to the developing embryo and is considered to be of primary importance in seed development (Brink and Cooper, 1947; Vijayaraghavan and Prabhakar, 1984, Lopes and Larkins, 1993; Berger, 2003). Moreover, arrested embryos in developing seeds from an interploid cross can often be grown into plantlets when the embryos are isolated from the seeds and cultured *in vitro* (Sharma *et al.*, 1996), supporting the consideration that an imbalanced parental genome disturbs the development of endosperm more severely than that of a zygote or embryo. However, success in embryo rescue is often dependent on the direction of crossing. For example, rice 2m:1p embryos, which were isolated from the 4*x*×2*x* crossed developing seeds, grew into plantlets via tissue culture; however, no successful embryo rescue was observed when 1m:2p embryos isolated from the 2*x*×4*x* crossed developing seeds were cultured (Sekine *et al.*, 2013). These results suggest that an imbalance of the parental genome may affect the development of zygotes as well as endosperms. However, the precise effect of an imbalance of the parental genome in zygotic development and embryogenesis has not been addressed, because it is difficult to reproduce the parental genome-imbalanced situation in zygotes and to observe the precise developmental profiles of such zygotes without the influence of endosperm, which is tissue neighboring the zygote/embryo.

Herein, in order to address distinctly whether zygotic development is dependent on the balance of the parental genome, we produced a polyploid zygote with an imbalanced parental genome ratio by *in vitro* fertilization using isolated rice gametes (Uchiumi *et al.*, 2007) and observed the developmental profiles of such polyploid zygotes by single cell culture. Most polyploid zygotes with an excess maternal gamete/genome developed normally as diploid zygotes, whereas approximately half to three-quarters of polyploid zygotes with a paternal excess showed developmental arrests. These results suggested that parental genomes are synergistically utilized in zygotes with different functions.

## Materials and methods

### Plant material and isolation of gametes

*Oryza sativa* L. cv. Nipponbare plants were grown in an environmental chamber (K30-7248; Koito Industries, Yokohama, Japan) at 26 °C under a 13 h light/11 h dark photoperiod. Transformed rice plants expressing the histone H2B–green fluorescent protein (GFP) fusion protein were prepared as previously described (Abiko *et al.*, 2013). The isolation of egg cells and sperm cells from rice flowers was conducted as described (Uchiumi *et al.*, 2006).

### Production of diploid zygotes and polyploid zygotes with imbalanced parental gametes, and zygote culture

**Fig. 1.**
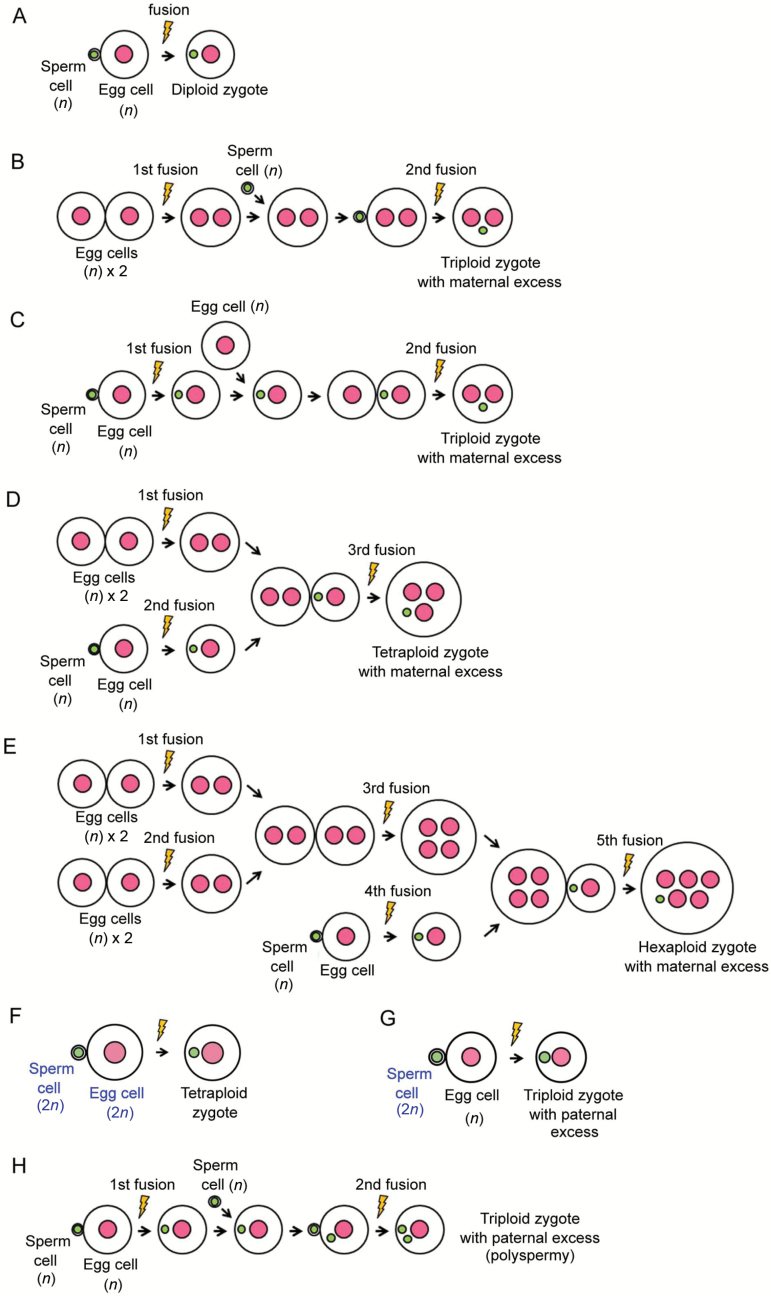
Schematic illustrations of the procedures to produce a polyploidy zygote by *in vitro* fusion. (A) Procedure to produce a diploid zygote. (B) One procedure to produce a maternal-excess triploid zygote. Two egg cells were fused at the first fusion, and the fusion product of two egg cells was further fused with a sperm cell to produce a triploid zygote of 2m:1p (second fusion). (C) Another procedure to produce a maternal-excess triploid zygote. An egg cell was first fused with a sperm cell, and the resulting zygote was fused with a second egg cell to produce a triploid zygote of 2m:1p (second fusion). (D) Procedure to produce a tetraploid zygote with a maternal excess. Two egg cells were fused at the first fusion. At the second fusion, an egg cell was fused with a sperm cell to produce a diploid zygote. After the second fusion, the fusion product of two egg cells was fused with the diploid zygote, resulting in the production of a tetraploid zygote of 3m:1p (third fusion). (E) Procedure to produce a maternal-excess hexaploid zygote. Two sets of fusion products of two egg cells were prepared at the first fusion and the second fusion. After the second fusion, the two fusion products were fused at the third fusion. At the fourth fusion, an egg cell was fused with a sperm cell to produce a diploid zygote. After the fourth fusion, the diploid zygote was fused with a fusion product produced by the third fusion to produce a hexaploid zygote of 5m:1p (fifth fusion). (F) Procedure to produce a balanced tetraploid zygote (2m:2p) by the fusion of a diploid egg cell with a diploid sperm cell. (G) Procedure to produce a paternal-excess triploid zygote (1m:2p) by electro-fusion of an egg cell with a diploid sperm cell. (H) Procedure to produce a paternal-excess triploid (polysermic) zygote using an egg cell and two sperm cells (Toda *et al.*, 2016). Pink and green circles indicate the egg and sperm nuclei, respectively. Yellow indicates the point of electro-fusion.

Zygotes were prepared from gametes that were isolated from wild-type rice plants or transformed rice plants expressing H2B–GFP. To prepare a diploid zygote, an isolated egg cell and a sperm cell were electro-fused according to Uchiumi *et al.* (2007). Electro-fusion of the isolated gametes for producing a polyploid zygote was conducted as previously reported (Toda *et al.*, 2016) but with modifications. Schematic illustrations of the procedure to produce polyploid zygotes are shown in Fig. 1. To produce a maternal-excess triploid zygote (2m:1p), two egg cells were fused at the first fusion, and the fusion product of the two egg cells was further fused with a sperm cell (second fusion, Fig. 1B). Alternatively, an egg cell was first fused with a sperm cell, and the resulting diploid zygote was further fused with a second egg cell to produce a triploid zygote (Fig. 1C). For production of a maternal-excess tetraploid rice zygote (3m:1p), two egg cells were fused at the first fusion, and, at second fusion, an egg cell was fused with a sperm cell to produce a diploid zygote. After the second fusion, the fusion product of two egg cells was fused with the diploid zygote (third fusion) resulting in the production of a tetraploid zygote of 3m:1p (Fig. 1D). In the case of production of a maternal-excess hexaploid rice zygote (5m:1p), two sets of fusion products of two egg cells were prepared at the first fusion and the second fusion. After the second fusion, the two fusion products were fused at the third fusion. At the fourth fusion, an egg cell was fused with a sperm cell to produce a diploid zygote. After the fourth fusion, the diploid zygote was fused with the fusion product produced by the third fusion to produce a hexaploid zygote of 5m:1p (Fig. 1E). Polyspermic zygotes and paternal-excess triploid zygotes were produced by serial fusion of two sperm cells with an egg cell according to Toda *et al.* (2016). In addition to production of polyploid zygotes by repeated fusion of haploid gametes, a haploid gamete isolated from a diploid rice plant was electro-fused with a diploid gamete isolated from a tetraploid plant to produce a paternal-excess triploid zygote (Fig. 1G).

The resulting polyploid zygotes were cultured using a Millicell-based method to observe their development into two-celled embryos, globular-like embryos, cell masses, and white calli (Uchiumi *et al.*, 2007). Plantlets were regenerated from white calli as previously described (Toki *et al.*, 2006).

### Microscopical observations

Zygotes/embryos expressing H2B–GFP were observed under a BX-71 inverted fluorescence microscope (Olympus, Tokyo, Japan) with 460–490 nm excitation and 510–550 nm emission wavelengths (U-MWIBA2 mirror unit; Olympus). Digital images of gametes, zygotes, and their resulting embryos were obtained using a cooled charge-coupled device camera (Penguin 600CL; PixCera, Los Gatos, CA, USA) and InStudio software (PixCera).

### Flow cytometry analyses

To examine the ploidy level of plants regenerated from polyploid zygotes, the DNA content per nucleus was measured by flow cytometry using a CyFlow Ploidy Analyzer PA-II (Partec, Münster, Germany) and a CyStain UV Precise P Kit (Partec). For this analysis, fresh leaf material (5 mm^2^) was chopped with a sharp razor in 200 μl extraction buffer from the kit. Then, 1 ml staining solution from the kit was added to the chopped tissues, and they were stained for 1 min. The crushed tissue and buffer was filtered through a 30 μm nylon mesh (Partec), and the filtered samples were loaded into the ploidy analyzer. Approximately 1000–2000 nuclei were measured for each sample. Measurements were conducted twice for most samples. Diploid plants (2*n*=24, *Oryza sativa* L. cv. Nipponbare) were used as the control.

## Results

### Production and development of triploid zygotes with a maternal excess

**Fig. 2.**
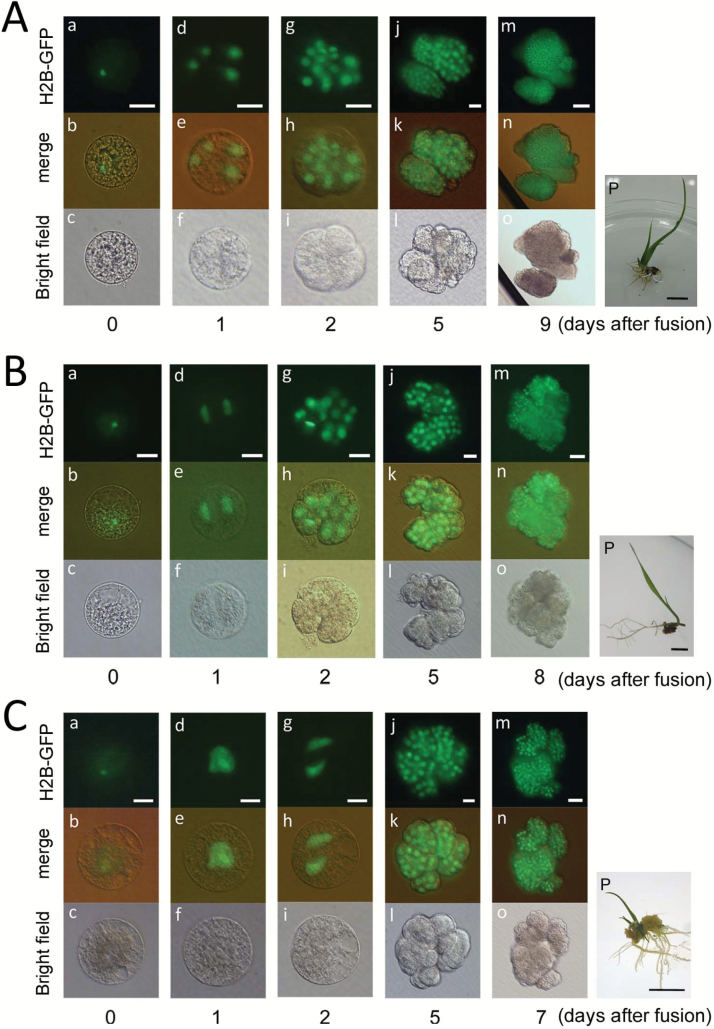
Development of triploid (A), tetraploid (B), and hexaploid (C) rice zygotes with an excess maternal genome. (A) A maternal-excess triploid zygote was produced by fusion of two egg cells with a sperm cell expressing H2B–GFP, and the resulting zygote was observed (a–c). The triploid zygote developed into a four-celled embryo at 1 d after fusion (d–f), a globular-like embryo at 2 d after fusion (g–i), and a cell mass at 5 and 9 d after fusion (j–o). The cell mass was regenerated into a plantlet (p). (B) A maternal-excess tetraploid zygote was produced by fusion of three egg cells with a sperm cell expressing H2B–GFP, and the resulting fused gametes were observed (a–c). The tetraploid zygotes developed into a two-celled embryo at 1 d after fusion (d–f), a globular-like embryo at 2 d after fusion (g–i), and a cell mass at 5 and 9 d after fusion (j–o). The cell mass was regenerated into a plantlet (p). (C) A hexaploid zygote was produced by fusion of five egg cells with a sperm cell expressing H2B–GFP, and the resulting fused gametes were observed (a–c). The zygote underwent the karyogamy process at 1 d after fusion (a–f) and developed into a two-celled embryo at 2 d after fusion (g–i) and a cell mass at 5 and 7 d after fusion (j–o). The cell mass was regenerated into a plantlet (p). Scale bars: 20 µm (Aa–l, Ba–l, Ca–l); 50 µm (Am–o, Bm–o, Cm–o); 1 cm (Ap, Bp, Cp). (This figure is available in color at *JXB* online.)

Diploid zygotes were produced by electro-fusion of an egg cell with a sperm cell (Fig. 1A). A maternal-excess triploid zygote was produced by the fusion of two egg cells of the wild-type with one sperm cell expressing H2B–GFP through two different procedures (Fig. 1B, C). In one procedure, two egg cells were fused at the first fusion, and the resulting fused cell was further fused with a sperm cell, resulting in a triploid zygote (Fig. 1B). In the other procedure, an egg cell was first fused with a sperm cell, and the resulting diploid zygote was further fused with a second egg cell to produce a triploid zygote (Fig. 1C). As shown in Fig. 2Aa–c, a triploid zygote with a sperm nucleus fluorescently labeled with H2B–GFP was successfully prepared. The triploid zygote developed into a four-celled embryo (Fig. 2Ad–f), a globular-like embryo consisting of approximately 14–16 cells (Fig. 2Ag–i), and then a cell mass (Fig. 2Aj–o). The time course for early development of the triploid zygote was equivalent to that of diploid zygotes (Uchiumi *et al.*, 2007). The cell mass further grew into a white callus, and the callus regenerated into a plantlet (Fig. 2Ap). Although the plants flowered, mature seeds hardly formed on these plants (Supplementary Table S1 at *JXB* online). The sterility of the plants is typical of triploid rice plantlets (Hu and Ho, 1963).

It has been reported that in the case of diploid zygotes, most of the zygotes produced *in vitro* grew into globular-like embryos, and white calli and plantlets were obtained efficiently from the diploid globular-like embryos (Table 1; Uchiumi *et al.*, 2007). In the study, the developmental profiles of 15 triploid zygotes were monitored, and all of the triploid zygotes developed into globular-like embryos such as those of diploid zygotes (Table 1). These results suggest that triploid zygotes with an excess maternal genome have the potential to develop as a diploid zygote, and that rice zygotes are tolerant to a double dose of maternal gametes/genomes.

**Table 1.** Developmental profiles of polyploid zygotes with unbalanced parental genome ratio

Parental balance	Ploidy	Gamete used for fusion	Fusion procedure	No. of produced zygotes	No. of zygotes developed into each growth stage					
					Karyogamy	Two-cell embryo	Globular-like embryo	Cell mass	Cell colony	Plantlet
Balanced	2*x*	Egg+sperm	Fig. 1A	18	18	18	18	18	17	17
	4*x*	Egg (2*n*)+sperm (2*n*)	Fig. 1F	14	13	13	13	12	12	12
Maternal excess	3*x*	Egg×2+sperm	Fig. 1B, C	15	15	15	15	14	14	14
	4*x*	Egg×3+sperm	Fig. 1D	13	13	13	13	10	9	7
	6*x*	Egg×5+sperm	Fig. 1E	12	11	11	11	11	11	6
Paternal excess	3*x*	Egg+sperm×2	Fig. 1H	34*	30*	19*	18*	15*	10*	6*
	3*x*	Egg+sperm (2*n*)	Fig. 1G	17	7	4	4	2	2	2

*Total number of cells or embryos determined by the present study and Toda *et al.* (2016).

### Production and development of tetraploid and hexaploid zygotes with a maternal excess

To reveal the relationship between zygotic development and parental imbalance, we further produced polyploid zygotes with a triple and quintuple dose of a maternal genome and monitored their development. For production of tetraploid zygotes with a 3m:1p ratio, three egg cells and a sperm cell were fused through three rounds of fusion as shown in Fig. 1D. A tetraploid zygote with a sperm nucleus fluorescently labeled with H2B–GFP was successfully prepared (Fig. 2Ba–c). The tetraploid zygote divided into a two-celled embryo (Fig. 2Bd–f), a globular-like embryo (Fig. 2Bg–i), and then a cell mass (Fig. 2Bj–o). All 13 tetraploid zygotes that were prepared in this study developed into a globular-like embryo (Table 1), and the time course for early development in the tetraploid zygote was equivalent to that in diploid zygotes (Fig. 2B; Uchiumi *et al.*, 2007). The cell mass further divided into a white callus, and the callus regenerated into a plantlet (Fig. 2Bp). When the plants were grown in our environmental chamber, in which diploid plants showed fertility of 43.2%, the fertility of tetraploid flowers was determined to be 5.3% (Supplementary Table S1).

Hexaploid zygotes (5m:1p) were produced by serial five rounds of fusion of five egg cells and a sperm cell (Fig. 1E). The hexaploid zygote completed karyogamy (Fig. 2Ca–f), and then the zygote developed into a two-celled embryo (Fig. 2Cg–i) and a cell mass (Fig. 2Cj–o). Among the 12 hexaploid zygotes produced in this study, 11 zygotes developed into a globular-like embryo (Table 1). The time course for early development in the hexaploid zygote was delayed by approximately 1–2 d when compared with that in diploid, triploid, and tetraploid zygotes (Fig. 2Ag–i, Bg–i, Cg–i). This delay may be due to the large cell and genome volumes in the hexaploid zygote and its daughter cells. The cell mass from the hexaploid zygote further divided into a white callus, and the callus regenerated into a plantlet (Fig. 2Cp). The fertility of hexaploid flowers was determined to be approximately 0.3% (Supplementary Table S1).

### Ploidy level and flower size of plants derived from maternal-excess polyploid zygotes

**Fig. 3.**
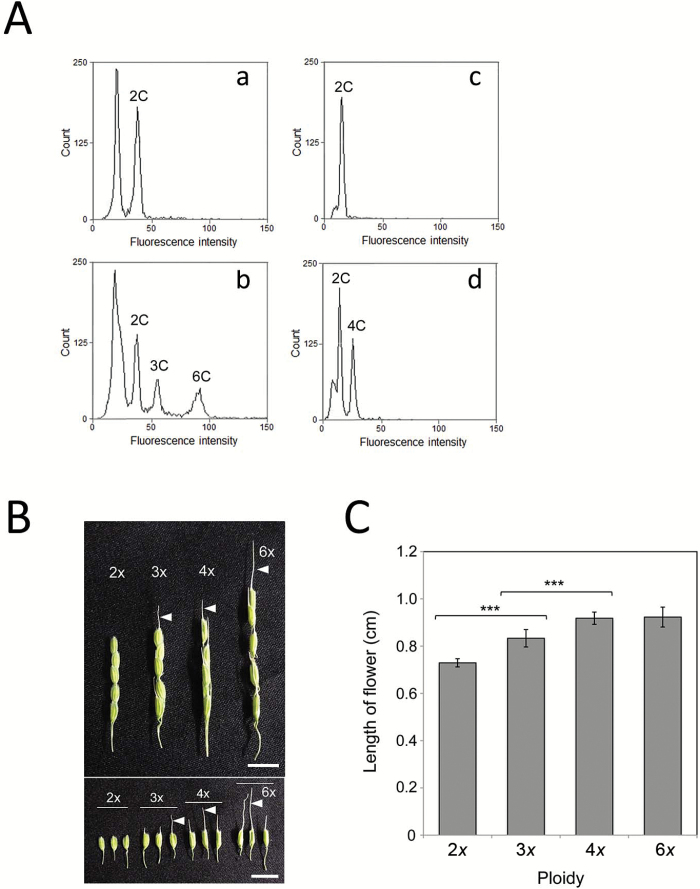
Ploidy level of possible polyploid rice plants derived from zygotes with a maternal excess (A) and flower size of polyploid rice plants (B, C). (A) Ploidy levels of rice plants regenerated from maternal-excess polyploid zygotes. After nuclei were extracted from the leaves of wild-type rice plants (a, c) or from the leaves of wild-type rice plants and plants regenerated from triploid and hexaploid zygotes (b) or tetraploid zygotes (d), the DNA content per nucleus was measured by flow cytometry. (B) Flowers of rice plants regenerated from polyploid zygotes. The flowers from wild-type rice plants (2*x*) are presented as a control. (C) Length of flowers harvested from diploid and maternal-excess polyploid rice plants. The data are mean ±SD of 14–23 flowers of diploid, triploid, tetraploid, and hexaploid rice plants. Arrowheads in (B) indicate awns. Asterisks in (C) indicate significant differences between diploid and triploid flowers and triploid and tetraploid flowers (Student’s *t*-test): ***P<0.001. Scale bars: 1 cm. (This figure is available in color at *JXB* online.)

When nuclei were extracted from the leaves of wild-type rice plants and the DNA content per nucleus was measured by flow cytometry, a single peak of 2C was detected (Fig. 3Aa, c). In the case of measurement of nuclei from the leaves of wild-type rice plants and plants regenerated from triploid and hexaploid zygotes, the peaks corresponding to 3C and 6C levels were detected in addition to a 2C peak (Fig. 3Ab), although the 6C peak was detected at a slightly lower intensity level than the calculated value. Similarly, nuclei from the leaves of wild-type rice plants and plants regenerated from tetraploid zygotes were prepared, and a peak corresponding to the 4C level was detected in addition to a 2C peak (Fig. 3Ad). These results suggested that the DNA content per nucleus was conserved in the plants regenerated from polyploid zygotes.

The size of flowers from plantlets regenerated from polyploid zygotes was larger in accordance with the increase of ploidy level between diploid and tetraploid, and flowers of polyploid plants formed well-developed awns (Fig. 3B, C).

### Production and development of polyspermic triploid zygotes

**Fig. 4.**
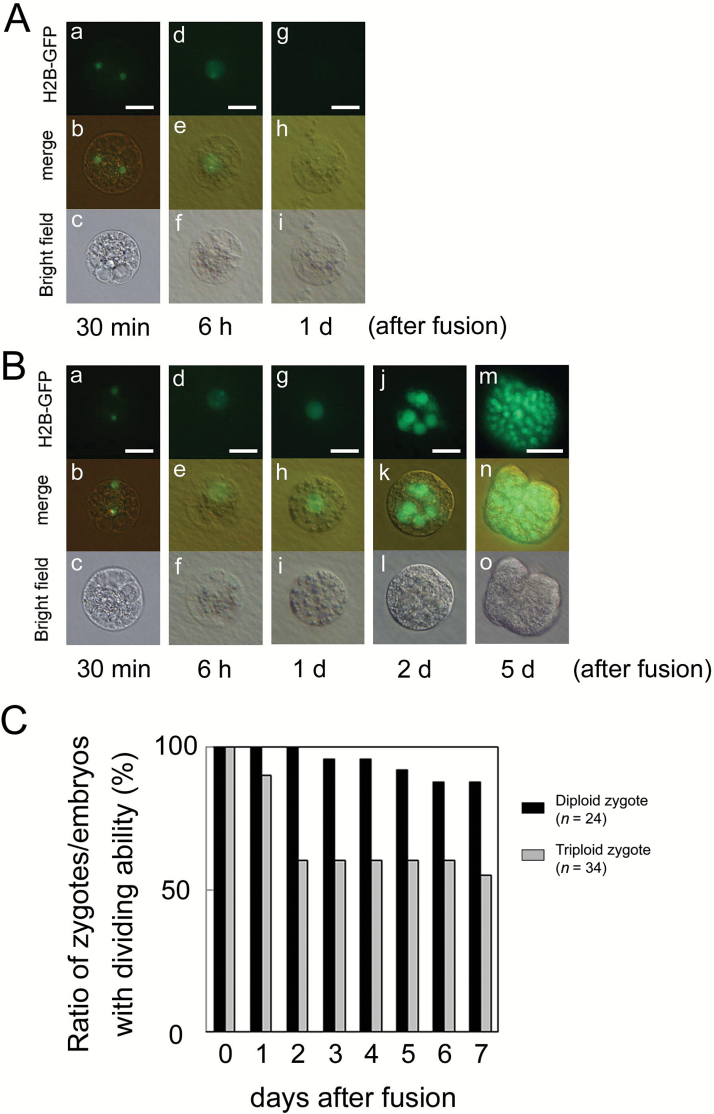
Developmental profiles of polyspermic triploid zygotes. An egg cell was serially fused with two sperm cells expressing H2B–GFP, and the resulting zygote was observed. (A) Failure of the first cell division in polyspermic zygote. Two sperm nuclei fluorescently labeled with H2B–GFP were observed in the fused egg cell (a–c). At 6 h after fusion, the H2B–GFP signal was detected in the zygotic nucleus, which was possibly derived from fusion of two sperm nuclei with an egg nucleus (d–f). However, the zygote did not divide at 1 d after fusion (g–i), and it degenerated thereafter. (B) In the polyspermic zygote, karyogamy was progressed to form a triploid zygotic nucleus (a–i), and the zygote further developed into a globular-like embryo at 2 d after fusion (j–l), and a cell mass (m–o). (C) Changes in rate of zygotes/embryos possessing dividing ability during culture of diploid zygotes (*n*=24) and polyspermic triploid zygotes (*n*=34). Upper, middle, and lower panels in (A, B) are fluorescence, merged fluorescence/bright-field, and bright-field images, respectively. Scale bars: 20 μm. (This figure is available in color at *JXB* online.)

We previously produced polyspermic rice zygotes using one egg cell and two sperm cells as in Fig. 1H, and it was suggested that approximately half of the zygotes developed normally; however, some portion of the produced zygotes showed developmental defects (Toda *et al.*, 2016). To verify the possibility of developmental arrest of polyspermic zygotes, an additional 20 polyspermic zygotes were produced in this study, and their developmental profiles were observed. As presented in Table 1, among a total of 34 polyspermic zygotes, four zygotes failed in karyogamy progression. Notably, after karyogamy, 11 out of 30 zygotes did not divide into a two-celled embryo (Fig. 4A; Table 1), indicating a defect in the first cell division. Although approximately one-third to half of the produced polyspermic zygotes showed abnormal development, most of the two-celled embryos from the remaining polyspermic zygotes further developed into a globular-like embryo, cell masses (Fig. 4B, C), and then regenerated into plantlets (Table 1). This suggests that polyspermy affects the early developmental step of zygotes but not the developmental profile of early embryos after first cell division of zygotes.

### Isolation of diploid gametes from tetraploid plants and *in vitro* fusion using the diploid gametes

Possible diploid egg cells and sperm cells were isolated from the tetraploid plants, which were regenerated from a tetraploid zygote produced by fusion of three egg cells with a sperm cell (Supplementary Fig. S1A, B). After electric fusion of these possible diploid gametes expressing H2B–GFP, male and female nuclei were detected in the zygote (Supplementary Fig. S1C–E). The zygote developed into a two-celled embryo, a globular-like embryo, a cell mass, and then a white callus (Supplementary Fig. S1F–O). From the white callus, multiple shoots were regenerated and plantlets were obtained (Supplementary Fig. S1P, Q). The ploidy level of the mature plants that regenerated from the zygotes was determined to be 4C by flow cytometry (Supplementary Fig. S1R, S). These results indicated that the plants regenerated from the zygotes were tetraploid as expected and that the gametes isolated from tetraploid plants can be used as diploid gametes for further analyses.

### Production and development of triploid zygotes with an excess paternal genome using haploid and diploid gametes

**Fig. 5.**
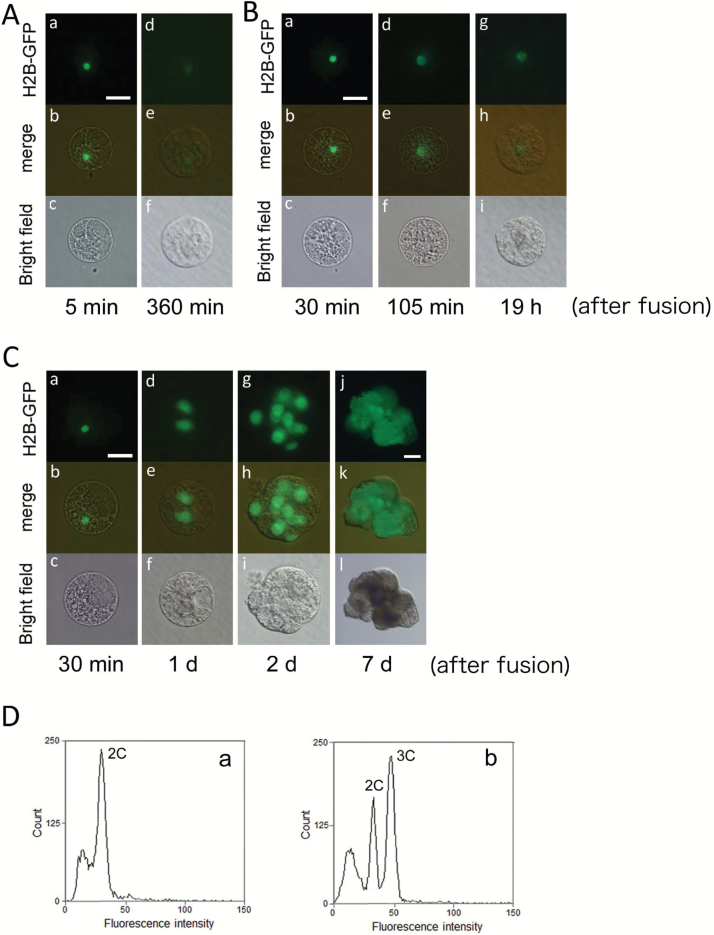
Developmental profiles of paternal-excess triploid zygotes produced by fusion of a haploid egg cell with a diploid sperm cell isolated from a tetraploid plant expressing H2B–GFP. (A) A sperm nucleus was detected in the zygote at 5 min and 360 min after fusion (a–f), and the zygote degenerated thereafter. (B) A sperm nucleus fluorescently labeled with H2B–GFP was detected in the zygote (a–c; 30 min after fusion). Although the sperm chromatin in zygotes decondensed in the fused nucleus during karyogamy (d–f; 105 min after fusion), no first cell division was observed at 19 h after fusion (g–i), and the zygote degenerated thereafter. (C) A sperm nucleus fluorescently labeled with H2B–GFP was detected in the zygote (a–c). Through possible karyogamy, the zygote developed into a two-celled embryo at 1 d after fusion (d–f), a globular-like embryo at 2 d after fusion (g–i), and a cell mass at 7 d after fusion (j–l). (D) Ploidy levels of rice plants regenerated from the paternal-excess triploid zygotes. After nuclei were extracted from the leaves of wild-type rice plants (a) or from the leaves of wild-type rice plants and plants regenerated from triploid zygotes (b), the DNA content per nucleus was measured by flow cytometry. Scale bars: 20 µm (A, B, Ca–i); 50 µm (Cj–l). (This figure is available in color at *JXB* online.)

Seventeen triploid zygotes with a 1m:2p genome ratio were prepared by fusion of a haploid egg cell with a diploid sperm cell (Fig. 1G). Among these 17 zygotes, nuclear fusion was not observed in 10 zygotes (Fig. 5Aa–f; Table 1), suggesting a defect in karyogamy progression. Although the remaining seven zygotes completed karyogamy, three zygotes did not divide and degenerated (Fig. 5Bg–i; Table 1). The remaining zygotes developed into a two-celled embryo (Fig. 5Ca–f), a globular-like embryo (Fig. 5Cg–i), and then a cell mass (Fig. 5Cj–l). The cell mass divided into a white callus, and then the white callus regenerated into a plantlet. The ploidy level of the mature plant regenerated from the possible triploid zygote was determined to be 3C (Fig. 5D).

### Asymmetric division of polyploidy zygotes with an excess maternal or paternal genome

**Fig. 6.**
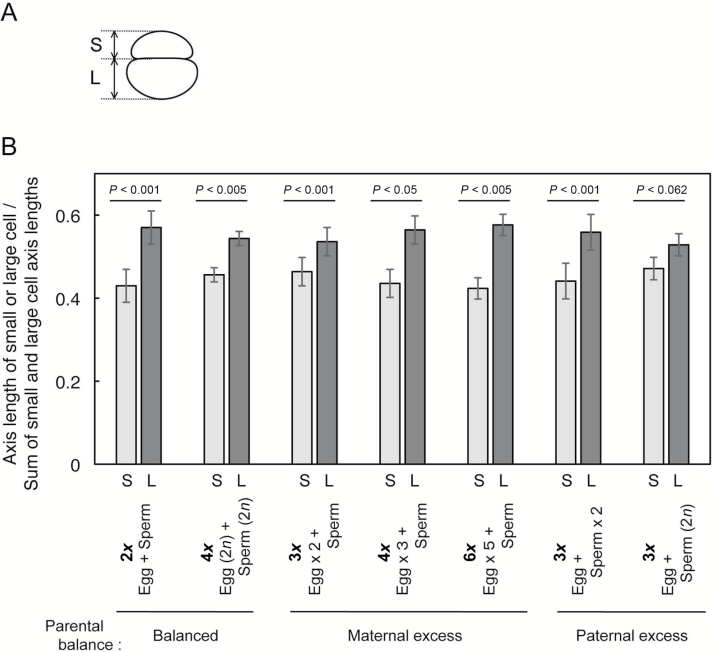
Asymmetric division of polyploid zygotes with maternal or paternal-excess genome. (A) An illustration showing how the axis lengths in a small cell (S) and a large cell (L) of a two-celled embryo were measured. (B) The ratio of axis length between a small cell (S) and a large cell (L) in a two-celled embryo. The data are mean ±SD of 20 diploid embryos, 7 balanced tetraploid embryos, 18 maternal-excess triploid embryos, 6 maternal-excess tetraploid embryos, 6 maternal-excess hexaploid embryos, 14 paternal-excess triploid embryos, 5 paternal-excess triploid embryos. Significant differences between S and L are shown in (B) (Student’s *t*-test).

Our previous study indicated that the diploid rice zygote produced *in vitro* divides asymmetrically into a two-celled embryo consisting of a small apical cell and a large vacuolated basal cell through possible reorganization of intracellular polarity in the developing zygote in a similar manner to the zygote in an embryo sac (Sato *et al.*, 2010). Therefore, to see if there was asymmetry in the two-celled embryos derived from polyploid zygotes, we measured the axis lengths of the small and large cells in the two-celled embryos, and the ratio of axis length between the small and the large cells was determined (Fig. 6A). The ratio of axis length between the small and the large cells in the two-celled embryo derived from diploid zygotes was determined as 0.43:0.57 (*n*=19) when the combined axis length of both cells was considered as 1 (Fig. 6B). As is the case with diploid zygotes, two-celled embryos from maternal-excess triploid, tetraploid, and hexaploid zygotes also showed asymmetry in cell size (Fig. 6B). In addition, asymmetric zygote division was also observed in paternal-excess triploid zygotes, although asymmetry in two-celled embryos from triploid zygotes that were produced by fusion of a haploid egg cell and a diploid sperm cell appeared to be small (Fig. 6B).

## Discussion

Polyploid rice zygotes with an excess maternal genome developed into asymmetric two-celled embryos and globular-like embryos, and most of these globular embryos grew into a callus and then a plantlet (Table 1). These results suggested that a double to quintuple dose of a maternal genome/genetic material in a zygote has no negative effect on the developmental profiles of zygotes. In contrast to a maternal excess in zygotes, our previous and present studies indicated that polyspermic rice zygotes, which were produced by serial fusion of an egg cell with two sperm cells, showed defects in zygotic development (Fig. 4; Table 1). Among the 34 polyspermic zygotes, 15 showed arrest in early zygotic development, although the remaining 19 zygotes mostly developed further into asymmetric two-celled embryos, globular-like embryos, and a callus structure (Table 1). Notably, the developmental profile of polyspermic zygotes was mainly disrupted at the step of first division of the zygote. These results suggest that an excess in the paternal genome/genetic material in zygotes triggers the developmental arrests. However, it remains unclear whether the developmental arrest in polyspermic zygotes is due to delivery of a double dose of genetic material from two sperm cells into an egg cell or the physiological shock of repeated and sequential electro-fusion of two sperm cells. Therefore, we next planned to produce paternal-excess triploid zygotes by single fusion of a haploid egg cell with a diploid sperm cell, which was isolated from tetraploid rice plants, and observe their developmental profiles. To check the cellular quality of diploid gametes, male and female gametes isolated from the flowers of tetraploid rice plants were fused, and the resulting possible tetraploid zygotes were cultured and regenerated. The results showed that possible tetraploid zygotes developed into early embryos, cell masses and plantlets, and they were determined to be tetraploid by flow cytometry (Supplementary Fig. S1), suggesting that gametes isolated from tetraploid plants can be used as diploid gametes. Therefore, paternal-excess triploid zygotes were prepared using pairs of haploid and diploid gametes.

Paternal-excess triploid zygotes produced by fusion of a haploid egg cell and a diploid sperm cell, termed E2S zygotes, showed arrests in zygotic development, as did the polyspermic triploid zygotes, termed ESS zygotes (Table 1). Interestingly, the majority of E2S zygotes showed defects in karyogamy in addition to the first cell division, although only a few ESS zygotes showed abnormality in karyogamy (Table 1). The difference in karyogamy progression between E2S and ESS zygotes may be explained by abnormal or excess activation of the fused egg cell, such as an inappropriate fertilization-induced increase of intracellular Ca^2+^ level (Denninger *et al.*, 2014; Hamamura *et al.*, 2014), which is triggered by immediate delivery of a double dose of cellular contents from a diploid sperm cell into an egg cell.

**Fig. 7.**
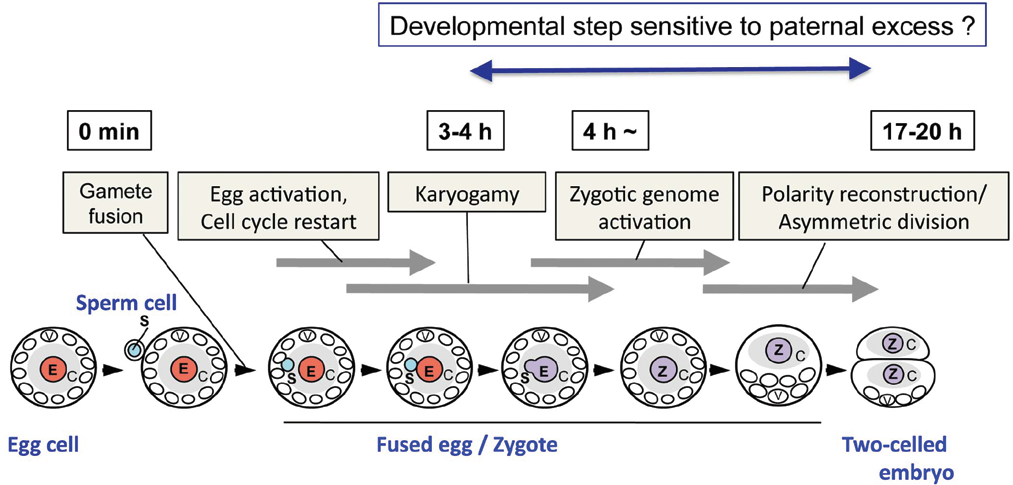
A schematic diagram of the progression of early development of rice zygote. After gamete fusion, the fused egg cell is activated via possible increase of intracellular calcium level as in fused egg cells of maize (Antoine *et al.*, 2001) and *Arabidopsis* (Denninger *et al.*, 2014; Hamamura *et al.*, 2014), and then karyogamy is completed 3–4 h after gamete fusion (Ohnishi *et al.*, 2014). *De novo* gene expression, termed zygotic genome activation, is initiated during or immediately after karyogamy (Ohnishi *et al.*, 2014), and then cellular polarity in zygote is reconstructed and the zygote asymmetrically divides into two-celled embryo consisting of small plasma-rich apical cell and large basal cell with developed vacuoles (Sato *et al.*, 2010). Excess of paternal materials in rice zygotes typically results in arrest at first cell division, suggesting the early developmental stage in rice zygote is sensitive step to excess of paternal genome. Light-blue and pink circles indicate sperm and egg nuclei, respectively. Violet circles indicate zygotic nuclei formed via karyogamy. C, cytoplasm rich region; E, egg nucleus; S, sperm nucleus; V, vacuoles Z, zygote nucleus.

Notably, developmental arrest after karyogamy was detected at the step of the first cell division of paternal-excess triploid zygotes. Approximately one-third of ESS zygotes (11 out of 30 zygotes) and of E2S zygote (3 out of 7 zygotes) could not divide and degenerated (Table 1). It has been indicated that the nascent synthesis of transcripts from zygotic parental genomes in zygotes is initiated either during karyogamy or within hours after fertilization in maize (Scholten *et al.*, 2002; Okamoto *et al.*, 2005; Meyer and Scholten, 2007), wheat (Sprunck *et al.*, 2005), Arabidopsis (Ueda *et al.*, 2011; Nodine and Bartel, 2012; Del Toro-De Leon *et al.*, 2014), and tobacco (Ning *et al.*, 2006; Zhao *et al.*, 2011). In rice zygotes, it has been reported that *de novo* zygotic gene expression, termed zygotic genome activation (ZGA), was initiated around 4 h after gamete fusion, during which karyogamy was mostly completed (Fig. 7; Ohnishi *et al.*, 2014). Thereafter, cellular polarity of the rice zygotes was reorganized, and the polarized zygotes asymmetrically divided into two-celled embryos at 17–20 h after gamete fusion (Sato *et al.*, 2010). This indicates that genes expressed in zygotes via ZGA will play important roles in zygotic development. The common developmental step that was disrupted in both E2S and ESS zygotes was the first cell division of the zygote, suggesting this developmental step is highly sensitive to an excess of the paternal genome (Fig. 7). Taken together, it is strongly suggested that ZGA-dependent gene expression profiles in zygotes might be partly disrupted by a parental genome imbalance, and subsequent zygotic development did not progress correctly.

Genes that are specifically or preferentially expressed from a paternal or maternal allele in zygotes or early embryos have been identified in Arabidopsis (Gehring *et al.*, 2011; Hsieh *et al.*, 2011; Raissig *et al.*, 2013), maize (Jahnke and Scholten, 2009; Waters *et al.*, 2011) and rice (Luo *et al.*, 2011). These possible parent of origin genes have been thought to play essential roles in the early development of zygotes and embryogenesis in angiosperms, although the mechanisms of regulation of expression of these genes and their gene functions still need to be explored (Luo *et al.*, 2014; Baroux and Grossniklaus, 2015; Zhao *et al.*, 2017). The present study provides direct evidence for the importance of parental balance in zygotes at the cellular level and for the possibility that genes that will be expressed specifically or preferentially from the paternal allele in early zygotes have a regulatory role in zygotic development. Investigations toward identification of such genes in rice zygotes are underway in our laboratories.

## Supplementary data

Supplementary data are available at *JXB* online.

Fig. S1. Isolated diploid rice gametes and development of tetraploid zygotes produced by *in vitro* fusion of diploid gametes.

Table S1. Fertility of rice plants derived from possible polyploid zygotes.

Supplementary Table S1

Supplementary Figure S1
