# Supplementary material for: An imbalanced parental genome ratio affects the development of rice zygotes
Source: J Exp Bot. 2018 Mar 10;69(10):2609–19. doi: 10.1093/jxb/ery094 (PMC5920335; doi:10.1093/jxb/ery094)
Supplement: Supplementary Table S1 [file ery094_suppl_supplementary_table_s1.pdf]

Supplementary l Table S1 Fertility of polyploid rice plants

Ploidy	No. of flowers	No. of mature seeds	Fertility (%)
Diploid	1,120	484	43.2
Triploid	1,083	0	0
Tetraploid	1,019	54	5.3
Hexaploid	287	1	0.3
