# Supplementary material for: An imbalanced parental genome ratio affects the development of rice zygotes
Source: J Exp Bot. 2018 Mar 10;69(10):2609–19. doi: 10.1093/jxb/ery094 (PMC5920335; doi:10.1093/jxb/ery094)
Supplement: Supplementary Figure S1 [file ery094_suppl_supplementary_figure_s1.pdf]

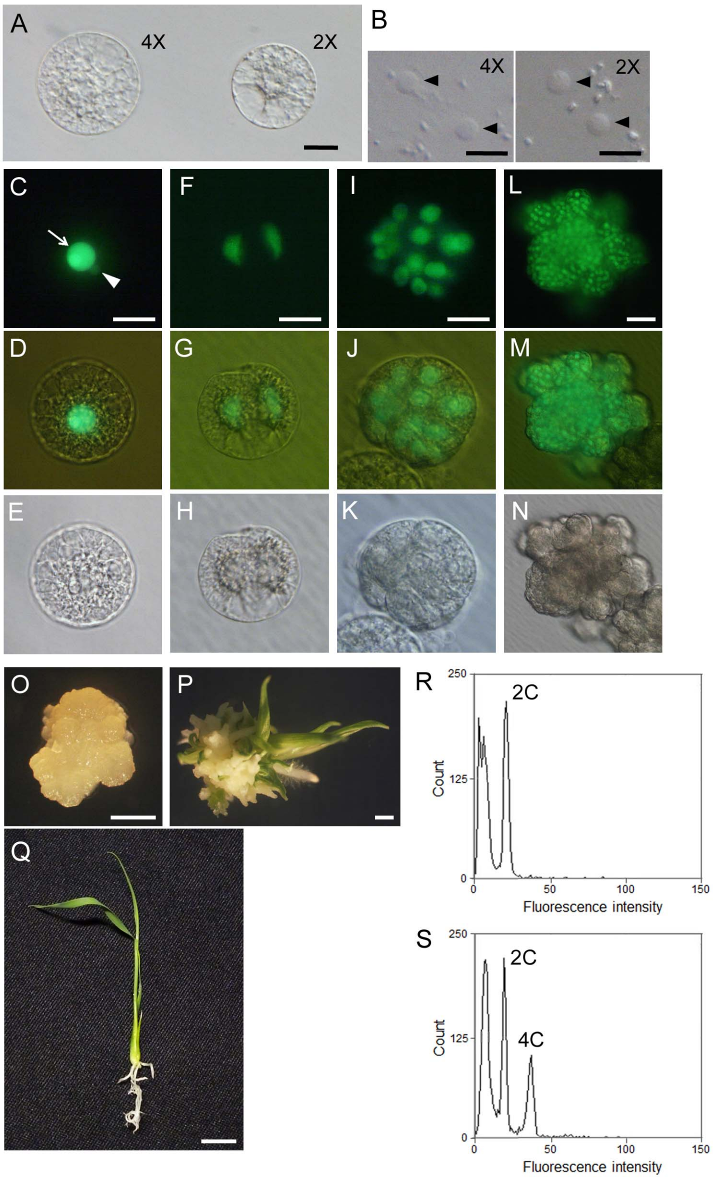

Supplementary Figure S1. Isolated diploid gametes (A and B) and the development of tetraploid rice zygotes produced by *in vitro* fusion of diploid gametes (C to S). A, Diploid and haploid egg cells isolated from tetraploid (4x) and diploid (2x) plants, respectively. B, Diploid and haploid sperm cells isolated from tetraploid (4x) and diploid (2x) plants, respectively. C to Q, The zygote (C to E) developed into a two-celled embryo (F to H), a globular-like embryo (I to K), a cell mass (L to M), and a white callus (O) during culture in liquid N6D medium. When the white callus was subsequently cultured on solid regeneration medium, it formed multiple shoots (P), and then plantlets were obtained (Q). R and S, Ploidy levels of rice plants

regenerated from the paternal excess triploid zygotes. After nuclei were extracted from the leaves of wild-type rice plants (R) or from the leaves of wild-type rice plants and plants regenerated from triploid zygotes (S), the DNA content per nucleus was measured by flow cytometry. Panels C, F, I and L are fluorescent images. Panels D, G, J and M present merged bright-field and fluorescent images. Panels E, H, K and N are bright-field images. The arrowheads in B indicate sperm cells. The arrow and arrowhead in panel C indicate egg and sperm nuclei fluorescently labeled with H2B-GFP, respectively. Scale bars = 20 µm in A to K, 50 µm in L to M, 1 mm in O and P, and 1 cm in Q.
